# Treatment with senicapoc in a porcine model of acute respiratory distress syndrome

**DOI:** 10.1186/s40635-021-00381-z

**Published:** 2021-04-19

**Authors:** Asbjørn G. Petersen, Peter C. Lind, Anne-Sophie B. Jensen, Mark A. Eggertsen, Asger Granfeldt, Ulf Simonsen

**Affiliations:** 1 Department of Biomedicine, Aarhus University, Aarhus, Denmark; 2 Department of Clinical Medicine, Anesthesiology, Aarhus University Hospital, Aarhus, Denmark; 3 Department of Intensive Care, Aarhus University Hospital, Palle Juul-Jensens Boulevard 99 G304, 8200 Aarhus, Denmark

**Keywords:** Acute respiratory distress syndrome, Calcium-activated potassium channels of intermediate conductance, Senicapoc, Pig

## Abstract

**Background:**

Senicapoc is a potent and selective blocker of KCa3.1, a calcium-activated potassium channel of intermediate conductance. In the present study, we investigated whether there is a beneficial effect of senicapoc in a large animal model of acute respiratory distress syndrome (ARDS). The primary end point was the PaO_2_/FiO_2_ ratio.

**Methods:**

ARDS was induced in female pigs (42–49 kg) by repeated lung lavages followed by injurious mechanical ventilation. Animals were then randomly assigned to vehicle (*n* = 9) or intravenous senicapoc (10 mg, *n* = 9) and received lung-protective ventilation for 6 h.

**Results:**

Final senicapoc plasma concentrations were 67 ± 18 nM (*n* = 9). Senicapoc failed to change the primary endpoint PaO_2_/FiO_2_ ratio (senicapoc, 133 ± 23 mmHg; vehicle, 149 ± 68 mmHg). Lung compliance remained similar in the two groups. Senicapoc reduced the level of white blood cells and neutrophils, while the proinflammatory cytokines TNFα, IL-1β, and IL-6 in the bronchoalveolar lavage fluid were unaltered 6 h after induction of the lung injury. Senicapoc-treatment reduced the level of neutrophils in the alveolar space but with no difference between groups in the cumulative lung injury score. Histological analysis of pulmonary hemorrhage indicated a positive effect of senicapoc on alveolar–capillary barrier function, but this was not supported by measurements of albumin content and total protein in the bronchoalveolar lavage fluid.

**Conclusions:**

In summary, senicapoc failed to improve the primary endpoint PaO_2_/FiO_2_ ratio, but reduced pulmonary hemorrhage and the influx of neutrophils into the lung. These findings open the perspective that blocking KCa3.1 channels is a potential treatment to reduce alveolar neutrophil accumulation and improve long-term outcome in ARDS.

**Supplementary Information:**

The online version contains supplementary material available at 10.1186/s40635-021-00381-z.

## Background

Acute respiratory distress syndrome (ARDS) is a life-threating condition and a frequent cause of morbidity and mortality in patients admitted to intensive care units [1, 2]. The current treatment strategies focus on lung-protective ventilation and conservative fluid therapy [3]. Despite recent promising treatments for ARDS including dexamethasone and recombinant angiotensin converting enzyme 2 (ACE2) [4, 5], therapies directly targeting the underlying pathophysiology have to be developed [6].

Preclinical studies suggest that ion channels situated in the endothelial and epithelial cell layers of the lung play a crucial role in activation of an inflammatory response and fluid transport across the alveolar–capillary barrier [7–10]. The calcium-activated potassium channels of intermediate conductance (KCa3.1) are ion channels highly expressed in the epithelium and white blood cells [11–13]. We and others have identified the KCa3.1 channel as a key regulator of fluid transport and inflammatory processes [14–18]. Upon lung injury, these effects are believed to be due to (1) KCa3.1’s active role in maintaining a sufficiently negative membrane potential to drive chloride efflux and concomitant water into the alveoli and (2) controlling calcium signaling pathways related to leucocyte recruitment and the production of inflammatory chemokines and cytokines. These data suggest that blocking KCa3.1 activity could be a potential therapeutic strategy to treat ARDS.

Senicapoc (ICA-17043) is a potent blocker of KCa3.1 channels that was found to be safe and well-tolerated when advanced to a phase-3 clinical trial for sickle cell anemia [19–23]. In an effort to repurpose senicapoc for treatment of ARDS, we tested if senicapoc treatment could prevent the development of ventilator-induced lung injury in mice [24]. We found that a single dose of senicapoc improved gas exchange measured as PaO_2_/FiO_2_ ratio, improved lung compliance, diminished the pulmonary inflammatory response (e.g., reduced neutrophil recruitment and pro-inflammatory cytokine release) and protected against changes in the alveolar–capillary barrier permeability.

The need for repeated blood sampling and advanced mechanical ventilation can limit smaller animal models of ARDS. In comparison, a larger animal like pig holds several advantages [25]. Pigs are more similar to humans in terms of physiology and anatomy and makes continues measurements of systemic/pulmonary hemodynamics possible. In order to further advance our findings, we tested the efficacy of senicapoc in a porcine model of ARDS.

## Methods

### Animal preparation

Three-months-old female crossbred Landrace/Yorkshire/Duroc pigs (42–49 kg) were used for the study. Only female pigs were included as it is more difficult to place the urinary catheters on male animals, without introducing a surgical insult. The effects of hormones are considered minimal, as the animals are adolescents at this age.

Animals were fasted overnight with free access to water. In the morning, the animal was premedicated with an intramuscular injection of midazolam (1 mg/kg). Before placement of the IV access, an additional dose of midazolam (0.625 mg/kg), s-ketamine (6.25 mg/kg), and atropine (0.5 mg) was given intramuscularly. Anesthesia was induced with IV midazolam (25 mg) and s-ketamine (250 mg) and maintained with a continuous infusion of fentanyl (60 μg/kg/h) and propofol (5 mg/kg/h). No neuromuscular blocking agent was used. The animals were placed in a supine position, intubated (8.0 mm, Portex tube, Smiths Medical International, Hoersholm, Denmark) and volume-controlled ventilated (Evita XL, Dräger, Germany) with a tidal volume (Vt) of 10 mL/kg, a positive end-expiratory pressure (PEEP) of 5 cmH_2_O, a fraction of inspired oxygen (FiO_2_) of 0.4, and an inspiration:expiration (I:E) ratio of 1:2. The ventilation rate was adjusted to maintain PaCO_2_ between 5.5 and 6 kPa. For fluid therapy, a bolus of Ringer’s acetate (20 mL/kg) was given to the animals after placement of the IV access, followed by a maintenance rate of 15 mL/kg/h for the remainder of the experimental protocol. Low blood glucose (< 3.5 mmol/L) was treated with doses of glucose IV (500 mg/L) until normoglycemia was achieved.

### Surgical preparation and monitoring

A urinary catheter was placed in order to measure urine output. Next, sheaths were inserted by ultrasound into the external jugular vein and femoral artery (10F and 8F, respectively). A Swan-Ganz catheter (7.5F, Edwards Lifesciences, CA, USA) was inserted through the external jugular vein to monitor cardiac output, mean pulmonary arterial pressure (MPAP), central venous pressure, mixed venous oxygen saturation, and core temperature (Vigilance Monitor, Edwards Lifesciences, CA, USA). The following parameters were also monitored throughout the experiment: ECG, heart rate, mean arterial pressure (MAP), and arterial oxygen saturation.

All animals were allowed to stabilize for 30 min between the surgical preparation and experimental protocol. A priori determined exclusion criteria at baseline were: MAP < 45 mmHg, MPAP > 25 mmHg, PaO_2_ < 18 kPa, lactate concentration > 3 mmol/L.

### Experimental protocol

**Fig. 1:**
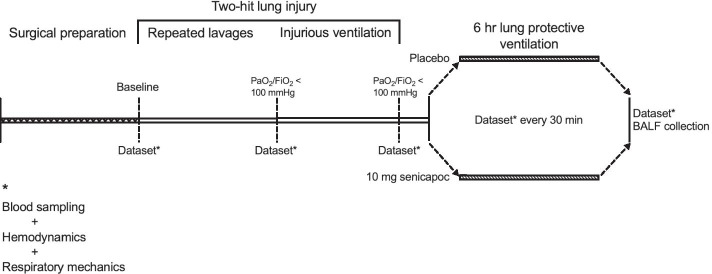
Schematic overview of the study design

After baseline physiological measurements, a two-hit lung injury encompassing repeated lung lavages and injurious mechanical ventilation was initiated (Fig. 1). This two-hit lung injury was developed to capture several features of human ARDS and aimed to fulfill the requirements listed by the American Thoracic Society [26]. Surfactant depletion by repeated lavages is known to cause severe hypoxemia (PaO_2_/FiO_2_ < 100 mmHg) and to decrease lung compliance. Repeated lavages, however, can be ineffective at producing injury to the alveolar–capillary barrier and cause neutrophilic infiltration into the lung [27]. Therefore, injurious mechanical ventilation was added as a secondary insult. Collectively, this induces a diffuse type of ARDS [28, 29].

FiO_2_ was increased to 1.0 and repeated lung lavages were applied. During each lavage, the lungs were filled with warm isotonic saline (37 °C, 30 mL/kg). Fluid was drained passively after 3 min of apnea or until MAP fell below 60 mmHg. Hereafter, the animal was reconnected to the ventilator and allowed to compensate (increase in MAP and oxygen saturation). Time between re-lavages did not exceed five minutes. Lung lavages were repeated until a PaO_2_/FiO_2_ < 100 mmHg was achieved. Then injurious mechanical ventilation using a peak inspiratory pressure (PIP) of 45 cmH_2_O, PEEP of 4 cmH_2_O and I:E of 1:1 were commenced. The respiratory rate was adjusted in an effort to maintain normocapnia. The injurious ventilation was continued until a PaO_2_/FiO_2_ of < 100 mmHg was achieved again. Animals requiring more than 4 h of injurious mechanical ventilation were excluded from the study.

Animals were randomized into two groups: (1) 10 mg senicapoc or (2) vehicle. Dosing was based on a previous senicapoc dose-escalation study [20, 21]. The primary investigators assessing all outcomes were blinded to group assignments. Thus, a neutral person from the technical staff was responsible for the randomization. The person drew a random note from a sealed non-opaque envelope. These notes were labeled either with the number “1” or “2”, corresponding to senicapoc or vehicle, respectively. Depending on the number drawn, solutions were prepared in a room away from the experimental site and containers of identical appearance were used. The total volume of the bolus was 12 mL and treatments were given IV over 15 min. A 0.1 M stock solution of senicapoc (MedChemExpress, Sweden) was prepared with DMSO and separated into aliquots. These were stored at − 80 °C. On each day of experimentation, one aliquot was diluted to a final concentration of 2.5% DMSO, 5% Cremophor EL and 92.5% saline. The same solution without senicapoc was used as vehicle. Concurrently with the start of infusion, a lung protective ventilatory protocol using BiPAP mode was initiated in order to achieve a defined oxygenation goal of a PaO_2_ between 8 and 10 kPa. Incremental FiO_2_/PEEP combinations were used for this purpose (see Additional file 5: Table S1) and the initial setting was a peak inspiratory pressure of 35 cmH_2_O, PEEP of 18 cmH_2_O, and I:E of 1:2. The respiratory rate was adjusted to maintain normocapnia, however, due the severity of the insult and ventilating the pigs within the limits of lung protective ventilation, the pigs developed hypercapnia. The driving pressure (= PIP minus PEEP) was set to be 17 cmH_2_O (i.e., if PEEP was reduced, PIP was also reduced to keep a constant driving pressure). Though this is higher than the recommended [30], pilots showed that this was necessary to keep the animals alive. Furthermore, pilot studies indicated that the protective ventilation remained close to the recommended Vt of 6 mL/kg. The lung protective ventilation was maintained and adjusted for 6 h. In this time period, blood gas analysis was performed every half-hour (ABL90 Flex Plus, Radiometer Medical, Denmark). Arterial blood samples were collected at every hour in the lung-protective ventilatory period. At the same time, dynamic (= Vt/PIP − PEEP) and static (= Vt/plateau pressure − PEEP) compliance were measured. Serum and plasma were isolated and stored at − 80 °C. Leucocyte count in the blood was obtained using an automated cell counter (Procyte Dx, Idexx, USA). At the end of the study, bronchoalveolar lavage (BAL) was performed and representative tissue samples were collected.

### BAL

Tracheostomy was performed by visual guidance, through which a fiberoptic bronchoscope (Olympus Medical Systems Corp, Japan) was advanced into the right lower lobe. Here, 60 mL of 37 °C saline was instilled over 15 s and immediately withdrawn. To calculate recovery, the total volume of the BAL fluid (BALF) was summed and the last 5 mL was analyzed on a cell counter. The remaining volume of the 5 mL was spun at 1000 *g* for 15 min at 4 °C and the supernatant was stored at − 80 °C.

### Vascular permeability

In order to examine protein permeability of the alveolar–capillary barrier, the total supernatant protein concentration of the BALF was determined spectrophotometrically (NanoDrop One^C^, Thermo Scientific, USA). Furthermore, the concentration of albumin, a high molecular weight plasma protein, was measured with a commercially available assay kit (Sigma-Aldrich, USA). From this, the BALF/plasma albumin ratio was calculated. Detection limit of the albumin assay was 0.1 mg/mL.

### Cytokine analysis

Concentrations of inflammatory cytokines (IL-1β, IL-6, IL-8, IL-10 and TNF-α) in the BALF and plasma were measured in duplicates with a multiplex assay (Procarta® Porcine Cytokine Assay Kit; Panomics, USA), according to the manufacturer’s instructions and as previously reported [31, 32]. Detection limits (pg/mL) were IL-1β (398), IL-10 (586), IL-6 (419), IL-8 (1606) and TNF-α (419).

### Histology

After finishing the BAL procedure, the thorax was opened and representative samples of the left lung lobes (top, middle and lower) were collected for histological analysis. Lung biopsies were collected from the left lung in order to avoid the influence of saline coming from the BAL procedure. The tissue specimens were immersed in 10% formalin for 48 h. Fixed lungs were embedded in paraffin, sectioned at 5-µm thickness, and stained with hematoxylin and eosin. At least 20 images per slide were obtained (EVOS M5000, Invitrogen), and histological assessment of the lung injury was performed on these. For scoring, the following parameters were considered for each image: (A) neutrophils in the alveolar space (none = score 0, 1–5 = score 1, > 5 = score 2); (B) neutrophils in the interstitial space (none = score 0, 1–5 = score 1, > 5 = score 2); (C) hyaline membranes (none = score 0, 1 = score 1, > 1 = score 2); (D) proteinaceous debris filling the airspaces (none = score 0, 1 = score 1, > 1 = score 2), and (E) alveolar septal thickening (< 2*x* = score 0, 2*x* − 4*x* = score 1, > 4*x* = score 2) [26]. Each independent variable was summed, weighted according to their relevance, and normalized to the number of fields ([(20 × A) + (14 × B) + (7 × C) + (7 × D) + (2 × E)]/number of fields × 100). The lung sections were examined for perivascular cuffs as a measure of liquid accumulation [14]. Measurement of hemorrhage in histological lung sections was assessed separately and performed as previously described [14]. In brief, areas containing aggregation of red blood cells in interstitial and alveolar space were measured using the image analysis software (ImageJ, NIH, Bethesda, MD, USA). All histological assessments were performed in a blinded fashion.

### Senicapoc plasma concentrations

A rapid, sensitive liquid chromatography-tandem mass spectrometry (LC–MS/MS) method was developed and validated for the quantification of total senicapoc plasma concentrations [33]. In brief, crude extracts were obtained and cleaned with methanol. Extracts were injected onto a Kinetex Biphenyl UHPLC column (1.7 μm, 2.1 mm I.D. × 100 mm) (Phenomenex, CA), and followed by analysis using a Sciex QTRAP 6500 + mass spectrometer with a TurboIonSpray probe for electrospray ionization. The lower limits of quantification were 0.1 µg/L.

### Statistical analysis

Data are presented as mean ± standard deviation (SD). Normality of data were investigated by inspecting *Q*–*Q* plots and data were logarithmically transformed when necessary in order to generate a Gaussian-distributed data set. Pairwise comparisons were done using Student’s *t*-tests. For continuous variables, a repeated-measurements ANOVA was used to analyze data for time/drug-dependent and between-group differences. A *P* < 0.05 was considered statistically significant. All statistical analyses were performed using GraphPad Prism 8 software (GraphPad Software, CA).

Nine animals were included in each group. This number was based on sample size calculations on the primary endpoint (PaO_2_/FiO_2_) from a separate pilot study (PaO_2_/FiO_2_: senicapoc: 140 ± 17, vehicle: 102 ± 17, *n* = 2 for both groups). A significance level of 5% and a power of 80% was assumed.

## Results

A total of 31 animals were included into the study. Thirteen animals were excluded according to the predefined protocol: four due to a high MPAP at baseline, four developed pneumothorax during injurious mechanical ventilation, and five exceeded the maximum 4 h of injurious mechanical ventilation. The remaining 18 animals were distributed as follows: senicapoc (*n* = 9), vehicle (*n* = 9). A single animal treated with senicapoc died from hypoxemia 15 min into the lung-protective ventilation protocol. No data from this animal were included in the analysis below. No differences were observed in body weight, number of lavages, duration of lavages and duration of injurious mechanical ventilation (Additional file 1: Fig. S1A–D).

### Hemodynamic and metabolic parameters

Changes in the main hemodynamic and metabolic parameters are shown in Additional file 6: Table S2. No differences between groups existed at baseline. In both groups, heart rate increased steadily over time and stabilized around 120 beats per minute. An initial peak in MAP after infusion of senicapoc or vehicle (MAP_1hr_: senicapoc, 105 ± 29 mmHg; vehicle, 96 ± 19 mmHg) was followed by decrease over time (MAP_6hr_: senicapoc, 69 ± 18 mmHg; vehicle, 68 ± 9 mmHg). MPAP increased two-fold after the two-hit lung injury and remained stable after this (MPAP_baseline_: senicapoc, 15 ± 2 mmHg; vehicle, 17 ± 5 mmHg / MPAP_6hr_: senicapoc, 29 ± 4 mmHg; vehicle, 29 ± 7 mmHg). No major changes were observed in cardiac output, blood glucose or lactate (Additional file 2: Fig. 2).

**Fig. 2:**
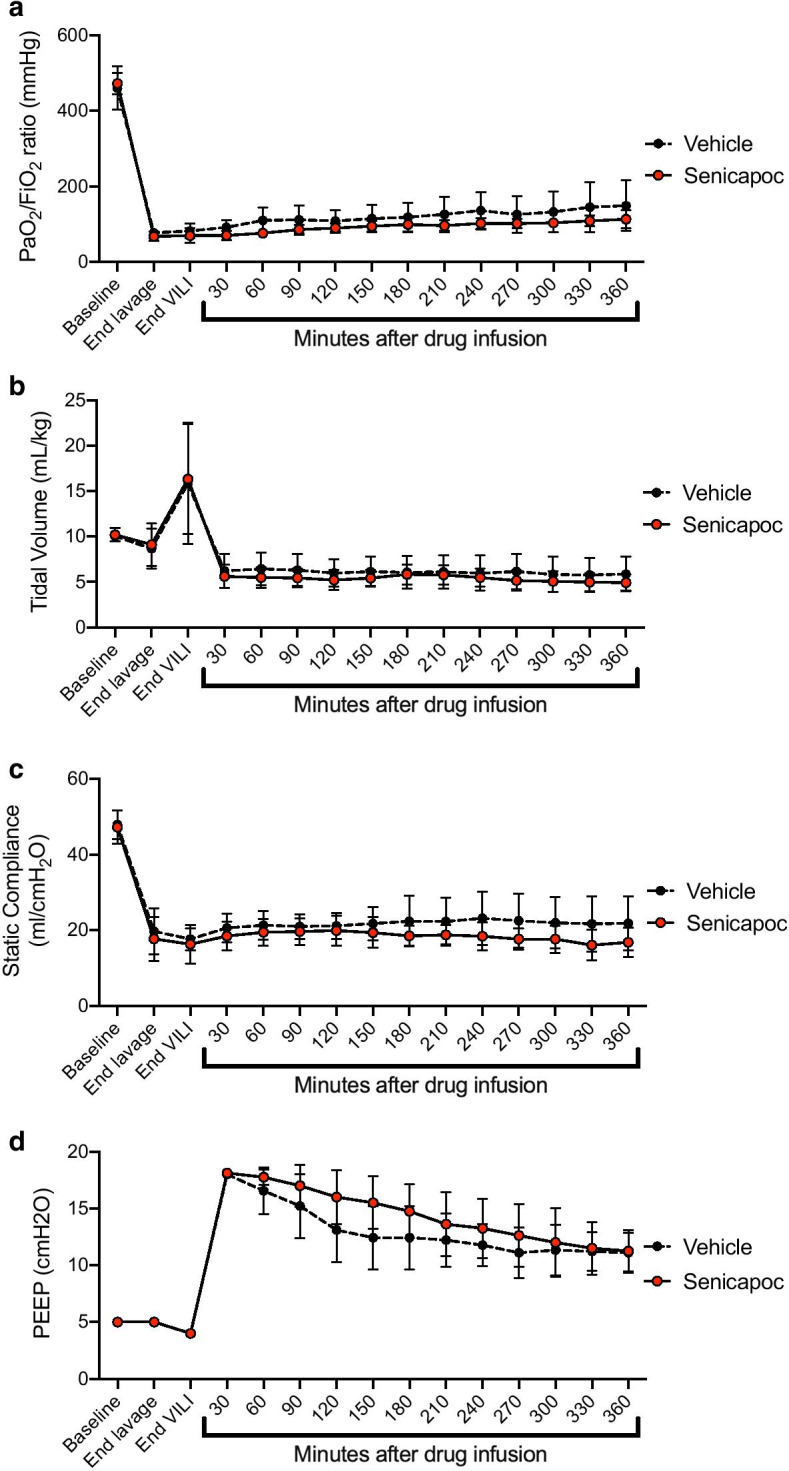
Administration of senicapoc did not improve lung function or respiratory mechanics. **a** PaO_2_/FiO_2_ ratio, **b** tidal volume, **c** static compliance and **d** PEEP. Data are presented as mean ± SD. Vehicle, *n* = 9 and senicapoc, n = 8. VILI, ventilator-induced lung injury

### Gas exchange and respiratory mechanics

The two-hit lung injury model markedly changed gas exchange and respiratory mechanics. As can be seen from Fig. 2a, PaO_2_/FiO_2_ ratio decreased from baseline during repeated lavages and injurious mechanical ventilation followed by a slow increase during the lung-protective ventilation. We observed no improvements in the final PaO_2_/FiO_2_ ratio when animals treated with senicapoc were compared to the vehicle group (senicapoc, 133 ± 23 mmHg; vehicle, 149 ± 68 mmHg; *P* = 0.93). Tidal volume and lung compliance (both static and dynamic) were also drastically reduced post-injury, yet no differences were found between the two experimental groups (Fig. 2b and c / Additional file 7: Table S3). PEEP, respiratory rate, minute volume and PIP were also unaffected by senicapoc-treatment (Fig. 2d / Additional file 7: Table S3).

### Albumin, inflammatory cells and cytokines in BALF

**Fig. 3:**
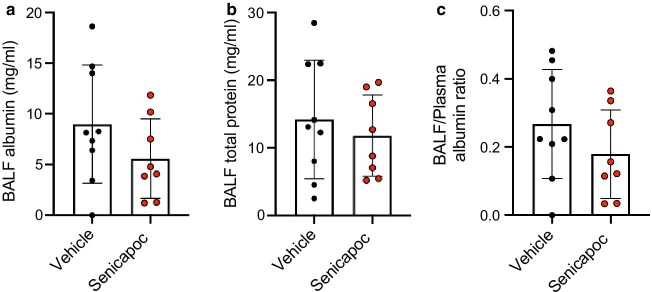
Administration of senicapoc did not improve alveolar–capillary barrier function. Measurements of **a** BALF albumin, **b** BALF total protein and **c** BALF/plasma albumin ratio. Data are presented as mean ± SD. Vehicle, *n* = 9 and senicapoc, *n* = 8. BALF, bronchoalveolar lavage fluid

Alteration in lung vascular and epithelial permeability was assessed by measuring the albumin concentration in the BALF. No significant reduction in albumin content was found compared with vehicle (Fig. 3a). This finding was supported by the total BALF protein concentrations as it remained unaffected by senicapoc treatment (Fig. 3b). When the BALF/plasma albumin ratio was calculated, no difference was found (Fig. 3c).

**Fig. 4:**
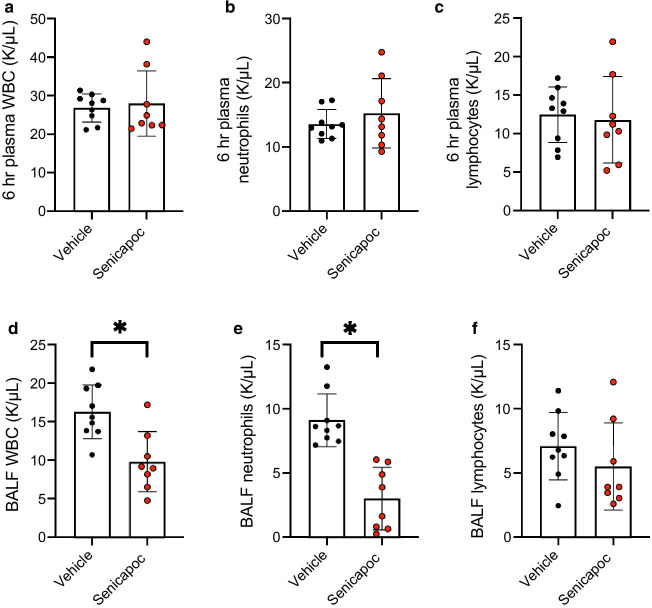
Administration of senicapoc reduces levels of BALF white blood cells and neutrophils. Cell counts of **a** plasma white blood cells at 6h; **b** plasma neutrophils at 6h; **c** plasma lymphocytes at 6h; **d** BALF white blood cells; **e** BALF neutrophils and **f** BALF lymphocytes. Data are presented as mean ± SD. *P < 0.05 for senicapoc against vehicle. Vehicle, *n* = 9 and senicapoc, *n* = 8. BALF, bronchoalveolar lavage fluid; WBC, white blood cells

Systemic white blood cells and the measured subtypes increased over time with no differences between the groups (Additional file 3: Fig. S3 / Fig. 4a–c). In BALF a significantly lower number of white blood cells and neutrophils (*P* < 0.05 for both variables) were found in animals treated with senicapoc compared to vehicle (Fig. 4d, e). Lymphocytes in BALF showed no statistical difference when compared between the two groups (Fig. 4f).

Levels of proinflammatory cytokines (IL-1β, IL-6, IL-8, and TNF-α) and anti-inflammatory cytokines (IL-10) in BALF and plasma are shown in Additional file 3: Fig. S3. A single dose of senicapoc was unable to change the cytokines levels measured in this study.

### Histological evaluation of lung injury

**Fig. 5:**
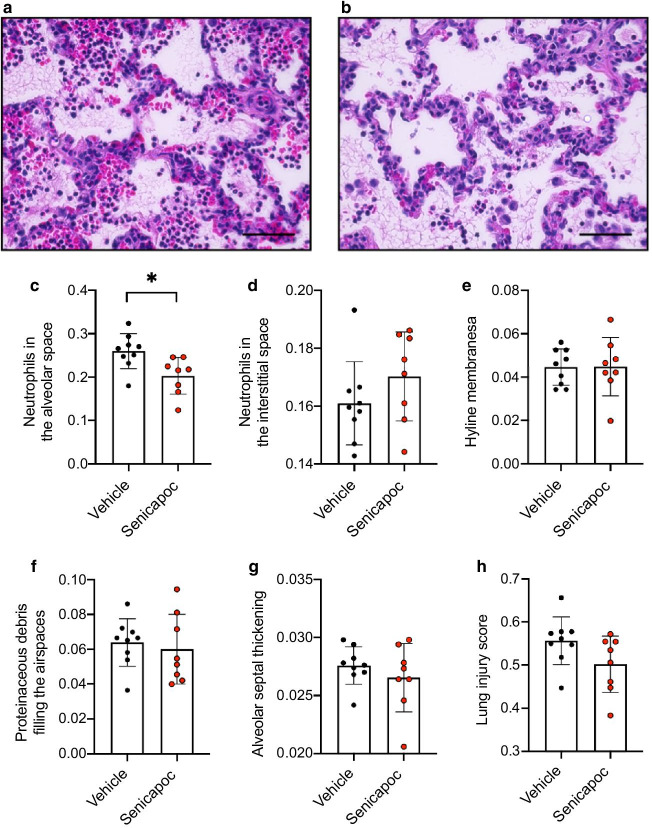
Senicapoc lowers alveolar neutrophil count, but it does not reduce lung injury score. Representative picture of **a** vehicle and **b** senicapoc. Individual scores of **c** neutrophils in the alveolar space, **d** neutrophils in the interstitial space, **e** hyaline membranes, **f** proteinaceous debris and **g** alveolar septal thickening, **h** cumulative lung injury score. Data are presented as mean ± SD. *P < 0.05 for senicapoc against vehicle. Vehicle, *n* = 9 and senicapoc, *n* = 8. The scale in picture A and B indicates 50 µm

**Fig. 6:**
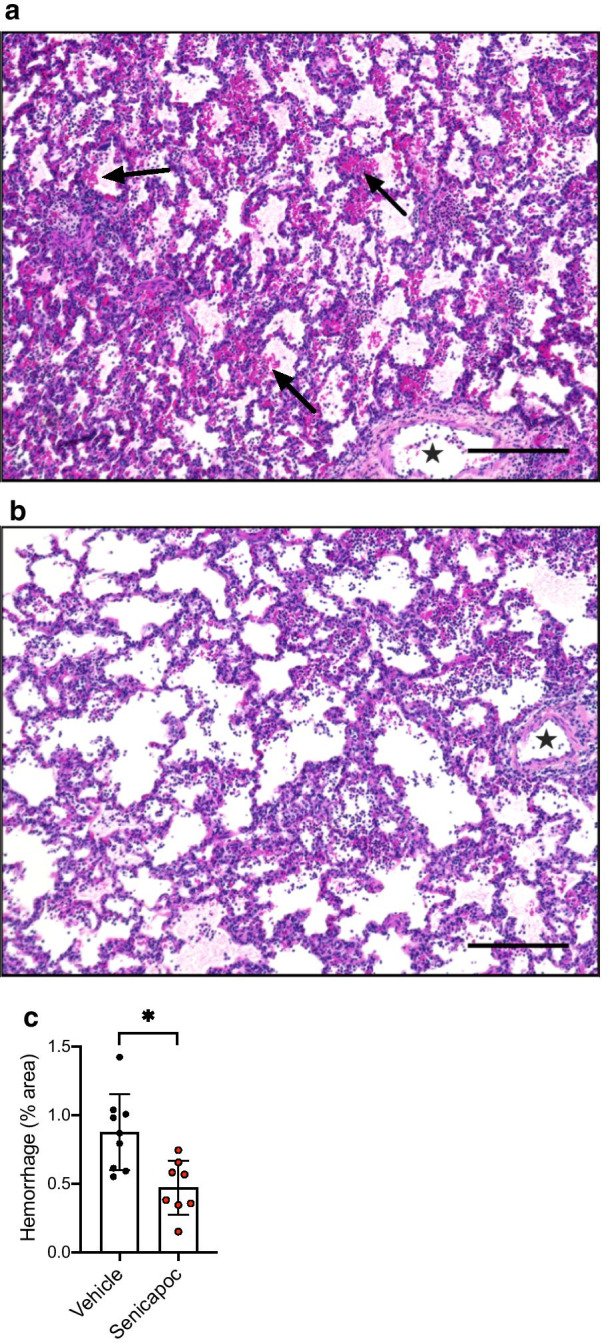
Senicapoc reduces histological pulmonary hemorrhage. Representative picture of **a** vehicle and **b** senicapoc. **c** Summary of interstitial and alveolar hemorrhage. Hemorrhage is indicated by black arrows. Note the absence of perivascular cuffs (filled star). Data are presented as mean ± SD. *P < 0.05 for senicapoc against vehicle. Vehicle, *n* = 9 and senicapoc, *n* = 8. The scale in picture A and B indicates 200 µm

Representative pictures of lung histology from senicapoc and vehicle-treated animals can be seen in Fig. 5a and b. Analysis revealed heavy infiltration of neutrophils into the alveolar space and the score was significantly lower for the senicapoc group (*P* < 0.05) (Fig. 5c). No change was observed for neutrophils in the interstitial spaces (Fig. 5d). Furthermore, prominent structural changes were visible with formation of hyaline membranes, signs of proteinaceous debris and alveolar septal thickening, but scoring remained insignificant between the two groups (Fig. 5e–g). Despite the reduced alveolar neutrophil level, the cumulative lung injury score of the senicapoc group was insignificant from the vehicle-treated animals (*P* = 0.08) (Fig. 5h). Examination of the lung sections from both experimental groups did not show perivascular cuffs as indication for liquid accumulation. Interstitial and alveolar hemorrhage was observed in both groups, but to a lesser extent in animals treated with senicapoc (*P* < 0.05) (Fig. 6a–c).

### Senicapoc plasma concentrations

Time course of senicapoc plasma concentration changes can be seen in Additional file 4: Figure S4. After 6 h of lung protective ventilation, a final mean concentration of 67 ± 18 nM (*n* = 8) was reached. The EC50 of senicapoc for the KCa3.1 channel is 11 nM [23, 34]. Therefore, these findings suggest that 10 mg senicapoc IV blocked the KCa3.1 channels 100% throughout the 6-h study period. All measurements on plasma from the vehicle group were below detection limit.

## Discussion

In the present study, we investigated whether senicapoc, a potent channel blocker of KCa3.1 channels, mitigates the harmful features of ARDS in a porcine animal model. To this aim, we used a two-hit lung injury model combining repeated lung lavages and injurious mechanical ventilation. A single senicapoc dose given IV was unable to improve the primary endpoint PaO_2_/FiO_2_ or the secondary endpoints, including alveolar–capillary barrier function, pulmonary inflammation, and overall lung injury score. However, the number of neutrophils in the BALF from animals treated with senicapoc was found to be significantly reduced and this was supported by the histological analysis. Also, pulmonary hemorrhage was diminished by senicapoc.

In our previous study using a murine model of ventilator-induced lung injury, senicapoc given by intraperitoneal injection was able to improve oxygenation measured as PaO_2_/FiO_2_ [24]. Conversely, we were unable to show a beneficial effect of senicapoc in the current study. This difference may be ascribed to the different animal models used, e.g., in contrast to the mice model no perivascular cuffs were observed in the porcine ARDS model in the present study. As human ARDS often have a multifactorial origin, a two-hit lung injury model is probably more reflective of the clinical situation where a single inciting stimulus can fail to capture the complex pathobiology seen in patients [26, 35]. Therefore, the porcine ARDS model can be a valuable translational model for drug evaluation, as an addition to rodent studies, before entering clinical trials, although one might also argue that the severity of lung injury produced was too severe for treatment and recovery.

Neutrophilic infiltration is a hallmark of human ARDS and a central driver for the inflammatory response [36–38]. Neutrophils have been found to constitute 68% of recovered lavage cells in ARDS patients [39]. Moreover, the neutrophil level in the lung correlates directly to the severity of abnormalities in gas exchange and the alveolar–capillary barrier and mortality from ARDS correlates with the extent of neutrophilia in the lung [38]. We have previously demonstrated that KCa3.1 inhibition by senicapoc was able to reduce the level of neutrophils in the BALF [24]. In the present study, our results agree with these findings as a significantly reduced number of neutrophils were observed in the BALF of senicapoc-treated animals. This was supported by a significant reduction in neutrophils in the alveolar space. As no differences in cytokine levels were found, the reduction may be related to KCa3.1’s role in cell migration [40]. KCa3.1 inhibition has been reported to drastically affect chemotactic and chemokinetic properties of mammalian neutrophils by impairing cell volume regulation [18]. These processes may be modulated by KCa3.1’s ability to regulate calcium entry. This will predominately affect calcium release-activated calcium (CRAC) channels, a protein important for activation of β_2_‐integrins and cytoskeletal rearrangement leading to polarization and migration in neutrophils [41, 42]. Although neutrophils are a vital part of the innate immune system where insufficient recruitment can lead to life-threatening infections, for example during bacterial pneumonia [43], the present findings suggest that senicapoc by inhibition of neutrophil migration may improve outcome of ARDS. Long-term studies will be required to investigate this effect.

As persistent elevation of inflammatory cytokines in the BALF of ARDS patients predicts a poorer outcome [44], therapeutic strategies targeting the inflammatory response could be an area of interest. Administration of senicapoc has previously been shown to reduce neuroinflammation as demonstrated by a downregulated production of pro-inflammatory mediators like TNF-α, IL-1β and IL-6 in isolated microglia cells [45–47]. In line with these findings, we observed a suppressed pulmonary inflammation in the lungs off senicapoc-treated mice following ventilator-induced lung injury with a reduction in IL-6, TNF-α, and the murine orthologs of IL-8 (KC and MIP-2) [24]. The present model did not show any effect on cytokine levels in either the BALF or plasma. This might question whether 6 h is enough to produce a cytokine response, though the marked increases in plasma leucocytes suggests otherwise. Furthermore, a study using a similar animal model also indicated early inflammation within four hours of observation [28].

In the initial exudative phase of ARDS, diffuse alveolar damage is a histological hallmark that is characterized by accumulation of white blood cells (predominantly neutrophils), hyaline membranes, and hemorrhage [48]. While diffuse alveolar damage can only be examined through postmortem biopsies, its presence has been associated with a higher mortality in ARDS patients [49]. The two-hit lung injury model presented several features of diffuse ARDS which was summarized in the applied histological injury scoring system. Here, we found no difference in the cumulative lung injury score. A separate histological analysis revealed a reduction of interstitial and alveolar hemorrhage by senicapoc; a finding in agreement with previous studies showing knockout of KCa3.1 channels protects against hemorrhage after TRPV4 activation [14]. This may indicate an effect of senicapoc on the permeability of the alveolar–capillary barrier, despite no observed perivascular cuffs and change in albumin concentrations.

### Limitations

One major limitation of this study is the short observation period of 6 h. This does not mimic the clinical scenario where ARDS patients admitted to the intensive care units generally require hospitalization for several weeks, depending on severity [1]. A longer observation period may have been beneficial for the present study’s primary endpoint. This is primarily because the resolution of the lung injury may have improved further in the senicapoc group due to a significantly lower neutrophil count in the BALF. Furthermore, the specific mechanism by which KCa3.1 plays a role in neutrophil migration remains to be elucidated, although this was not the primary scope of the present study. Further investigations on the effects of senicapoc should be investigated in animal models where the insult directly involves an augmented neutrophilic inflammatory response (e.g., endotoxin or live bacteria) or using a long-term ARDS animal model.

The lack of perivascular cuffs might indicate a lack of edema formation after injury. Though other parameters like hemorrhage and increased BALF albumin concentrations suggest changes to the permeability of the alveolar–capillary barrier, this is a limitation of the present model.

A positive effect of senicapoc on the permeability of the alveolar–capillary barrier was indicated by the hemorrhage analysis. The use of a transpulmonary thermodilution device would allow the measurements of pulmonary vascular permeability index and extracellular lung water where the latter is associated with ventilation/perfusion mismatch and pulmonary shunts [50]. Application of transpulmonary thermodilution would have helped to reinforce the results of the present study.

## Conclusion

In conclusion, there were no improvements in the primary endpoint PaO_2_/FiO_2_ with 10 mg senicapoc in a porcine animal model of ARDS. Our results support an important role of KCa3.1 in neutrophil migration. As the neutrophil level in the lung is an important determinant for the mortality of ARDS patients, senicapoc may have a future place in the treatment of these patients.

## Supplementary Information

**Additional file 1.**

**Additional file 2.**

**Additional file 3.**

**Additional file 4.**

**Additional file 5.**

**Additional file 6.**

**Additional file 7.**

## Data Availability

The datasets used and/or analyzed during the current study are available from the corresponding author on reasonable request.

## Abbreviations

ARDS: Acute respiratory distress syndrome
BAL: Bronchoalveolar lavage
BALF: Bronchoalveolar lavage fluid
I:E: Inspiratory–expiratory ratio
IV: Intravenous
KCa3.1: Calcium-activated potassium channels of intermediate conductance
MAP: Mean arterial pressure
MPAP: Mean pulmonary arterial pressure
PaO_2_/FiO_2_: Partial pressure of arterial oxygen to fraction of inspired oxygen ratio
PEEP: Positive end-expiratory pressure
