# Supplementary material for: Treatment with senicapoc in a porcine model of acute respiratory distress syndrome
Source: Intensive Care Med Exp. 2021 Apr 19;9:20. doi: 10.1186/s40635-021-00381-z (PMC8053424; doi:10.1186/s40635-021-00381-z)
Supplement: Supplementary file 5 — Additional file 5. [file 40635_2021_381_MOESM5_ESM.docx]

| **Table S1: Incremental FiO_2_/PEEP combinations.** | | | | | | | |
| --- | --- | --- | --- | --- | --- | --- | --- |
| FiO_2_ | 0.30 | 0.4 | 0.4 | 0.5 | 0.5 | 0.6 | 0.7 |
| PEEP | 5 | 5 | 8 | 8 | 10 | 10 | 10 |
|  |  |  |  |  |  |  |  |
| FiO_2_ | 0.7 | 0.7 | 0.8 | 0.9 | 0.9 | 0.9 | 1.0 |
| PEEP | 12 | 14 | 14 | 14 | 16 | 18 | 18 |
| FiO_2_, fraction of inspired oxygen; PEEP, positive end-expiratory pressure. | | | | | | | |
