# Supplementary material for: Treatment with senicapoc in a porcine model of acute respiratory distress syndrome
Source: Intensive Care Med Exp. 2021 Apr 19;9:20. doi: 10.1186/s40635-021-00381-z (PMC8053424; doi:10.1186/s40635-021-00381-z)
Supplement: Supplementary file 6 — Additional file 6. [file 40635_2021_381_MOESM6_ESM.docx]

| **Table S2: Hemodynamic and metabolic parameters** | | | | | | | | | | | | | | | **ANOVA (*P* =)** | | |
| --- | --- | --- | --- | --- | --- | --- | --- | --- | --- | --- | --- | --- | --- | --- | --- | --- | --- |
|  | Baseline | Post-injury | 30 min | 60 min | 90 min | 120 min | 150 min | 180 min | 210 min | 240 min | 270 min | 300 min | 330 min | 360 min | Group x Time | Time | Group |
| **Heart rate (BPM)** |  |  |  |  |  |  |  |  |  |  |  |  |  |  | 0.99 | **<0.05** | 0.68 |
| Senicapoc | 62 ±  14 | 103 ± 27 | 95 ± 14 | 121 ± 29 | 129 ± 40 | 139 ± 42 | 141 ± 35 | 138 ± 42 | 139 ± 30 | 132 ± 28 | 134 ± 26 | 131 ± 23 | 128 ± 21 | 127 ± 19 |  |  |  |
| Placebo | 61 ±  11 | 108 ± 39 | 99 ± 26 | 120 ± 26 | 127 ± 28 | 129 ± 29 | 128 ± 33 | 133 ± 35 | 135 ± 38 | 127 ± 36 | 131 ± 29 | 125 ± 28 | 121 ± 25 | 121 ± 27 |  |  |  |
|  | >0.99 | 0.78 | >0.99 | >0.99 | >0.99 | >0.99 | 0.99 | >0.99 | >0.99 | >0.99 | >0.99 | >0.99 | >0.99 | >0.99 |  |  |  |
| **Cardiac output (ml/min)** |  |  |  |  |  |  |  |  |  |  |  |  |  |  | **< 0.05** | 0.14 | 0.93 |
| Senicapoc | 4,6 ± 1,1 | 4,2 ± 0,8 | 5,3 ± 0,8 | 6,5 ± 1,3 | 6,5 ± 1,1 | 6,1 ± 1,7 | 5,8 ± 1,6 | 5,8 ±  1,6 | 6,1 ±  1,7 | 6,2 ± 1.7 | 5,9 ± 2,0 | 6,4 ± 2,1 | 6,1 ± 2,2 | 6,3 ± 2,4 |  |  |  |
| Placebo | 4,0 ± 1,0 | 3,2 ± 0,6 | 4,4 ± 1,0 | 5,0 ±  0,8 | 5,6 ± 1,0 | 5,7 ± 0,7 | 5,7 ± 0,7 | 5,6 ± 0,9 | 5,3 ± 0,9 | 5,4 ± 1,2 | 5,6 ± 1,1 | 5,7 ± 1,3 | 5,9 ± 1,3 | 5,6 ± 1,1 |  |  |  |
|  | 0.99 | 0.16 | 0.72 | 0.23 | 0.85 | >0.99 | >0.99 | >0.99 | 0.99 | 0.98 | >0.99 | 0.99 | >0.99 | >0.99 |  |  |  |
| **MAP (mmHg)** |  |  |  |  |  |  |  |  |  |  |  |  |  |  | 0.97 | **<0.05** | 0.94 |
| Senicapoc | 85 ±  14 | 76 ± 13 | 100 ± 23 | 105 ± 29 | 102 ± 33 | 86 ± 19 | 80 ± 19 | 77 ± 17 | 73 ± 20 | 74 ± 14 | 72 ± 16 | 70 ± 19 | 68 ± 18 | 69 ± 18 |  |  |  |
| Placebo | 83 ±  19 | 74 ± 10 | 100 ± 15 | 96 ± 19 | 94 ± 18 | 91 ± 21 | 87 ± 17 | 82 ± 6 | 76 ± 10 | 72 ± 12 | 73 ± 12 | 70 ± 10 | 70 ± 12 | 68 ± 9 |  |  |  |
|  | >0.99 | >0.99 | >0.99 | >0.99 | >0.99 | >0.99 | 0.99 | >0.99 | >0.99 | >0.99 | >0.99 | >0.99 | >0.99 | >0.99 |  |  |  |

| **Table S2 continued: Hemodynamic and metabolic parameters** | | | | | | | | | | | | | | | **ANOVA (*P* =)** | | |
| --- | --- | --- | --- | --- | --- | --- | --- | --- | --- | --- | --- | --- | --- | --- | --- | --- | --- |
|  | Baseline | Post-injury | 30 min | 60 min | 90 min | 120 min | 150 min | 180 min | 210 min | 240 min | 270 min | 300 min | 330 min | 360 min | Group x Time | Time | Group |
| **MAP_systolic_ (mmHg)** |  |  |  |  |  |  |  |  |  |  |  |  |  |  | 0.85 | **<0.05** | 0.56 |
| Senicapoc | 120 ±  13 | 118 ±  39 | 149 ±  28 | 156 ±  50 | 151 ±  49 | 125 ±  29 | 115 ± 23 | 108 ±  23 | 102 ±  31 | 110 ±  25 | 108 ±  27 | 105 ±  28 | 106 ±  24 | 88 ±  41 |  |  |  |
| Placebo | 117 ±  21 | 107 ±  12 | 148 ±  22 | 148 ±  40 | 145 ±  33 | 138 ±  34 | 135 ± 24 | 125 ±  15 | 117 ±  21 | 110 ±  26 | 105 ±  23 | 106 ±  23 | 106 ±  25 | 101 ±  17 |  |  |  |
|  | >0.99 | 0.99 | >0.99 | >0.99 | >0.99 | 0.99 | 0.79 | 0.79 | 0.99 | >0.99 | >0.99 | >0.99 | >0.99 | 0.99 |  |  |  |
| **MAP_diastolic_**  **(mmHg)** |  |  |  |  |  |  |  |  |  |  |  |  |  |  | 0.95 | **<0.05** | 0.65 |
| Senicapoc | 67 ±  12 | 60 ±  13 | 80 ±  27 | 87 ±  26 | 83 ±  32 | 67 ± 17 | 63 ± 18 | 65 ± 19 | 59 ± 21 | 56 ± 16 | 54 ± 17 | 51 ± 18 | 49 ± 17 | 51 ± 18 |  |  |  |
| Placebo | 66 ± 18 | 62 ± 13 | 77 ± 15 | 74 ± 19 | 72 ± 16 | 68 ± 18 | 66 ± 14 | 60 ± 5 | 57 ± 10 | 52 ± 13 | 54 ± 9 | 53 ± 8 | 50 ± 10 | 48 ± 11 |  |  |  |
|  | >0.99 | >0.99 | >0.99 | >0.99 | 0.99 | 0.99 | >0.99 | >0.99 | >0.99 | >0.99 | >0.99 | >0.99 | >0.99 | >0.99 |  |  |  |
| **MPAP (mmHg)** |  |  |  |  |  |  |  |  |  |  |  |  |  |  | 0.90 | **<0.05** | 0.25 |
| Senicapoc | 15 ± 2 | 31 ± 1 | 35 ± 8 | 36 ± 4 | 35 ± 4 | 34 ± 5 | 33 ± 4 | 30 ± 5 | 30 ± 5 | 31 ± 6 | 30 ± 5 | 30 ± 6 | 30 ± 5 | 29 ± 4 |  |  |  |
| Placebo | 17 ± 5 | 35 ± 9 | 40 ± 5 | 37 ± 5 | 38 ± 6 | 36 ± 3 | 33 ± 5 | 32 ± 4 | 33 ± 3 | 32 ± 7 | 32 ± 7 | 31 ± 5 | 30 ± 4 | 29 ± 7 |  |  |  |
|  | >0.99 | 0.78 | >0.99 | >0.99 | >0.99 | >0.99 | 0.99 | >0.99 | >0.99 | >0.99 | >0.99 | >0.99 | >0.99 | >0.99 |  |  |  |

| **Table S2 continued: Hemodynamic and metabolic parameters** | | | | | | | | | | | | | | | **ANOVA (*P* =)** | | |
| --- | --- | --- | --- | --- | --- | --- | --- | --- | --- | --- | --- | --- | --- | --- | --- | --- | --- |
|  | Baseline | Post-injury | 30 min | 60 min | 90 min | 120 min | 150 min | 180 min | 210 min | 240 min | 270 min | 300 min | 330 min | 360 min | Group x Time | Time | Group |
| **MPAP_systolic_ (mmHg)** |  |  |  |  |  |  |  |  |  |  |  |  |  |  | 0.34 | **<0.05** | **<0.05** |
| Senicapoc | 23 ± 4 | 33 ± 6 | 39 ± 5 | 39 ± 4 | 38 ± 4 | 37 ± 5 | 36 ± 7 | 33 ± 6 | 34 ± 5 | 35 ± 5 | 34 ± 4 | 34 ± 5 | 34 ± 5 | 32 ± 6 |  |  |  |
| Placebo | 24 ± 4 | 41 ± 10 | 48 ± 6 | 44 ± 7 | 45 ± 7 | 43 ± 5 | 40 ± 6 | 37 ± 6 | 38 ± 4 | 39 ± 9 | 38 ± 8 | 38 ± 5 | 37 ± 7 | 36 ± 6 |  |  |  |
|  | >0.99 | 0.55 | 0.10 | 0.80 | 0.18 | 0.38 | 0.96 | 0.94 | 0.90 | 0.99 | 0.97 | 0.96 | 0.99 | 0.90 |  |  |  |
| **MPAP_diastolic_**  **(mmHg)** |  |  |  |  |  |  |  |  |  |  |  |  |  |  | 0.50 | **<0.05** | 0.76 |
| Senicapoc | 9 ± 3 | 28 ± 8 | 31 ± 6 | 33 ± 5 | 32 ± 4 | 31 ± 4 | 31 ± 6 | 28 ± 7 | 27 ± 6 | 27 ± 7 | 25 ± 6 | 27 ± 8 | 26 ± 6 | 25 ± 5 |  |  |  |
| Placebo | 13 ± 7 | 30 ± 9 | 33 ± 6 | 31 ± 6 | 33 ± 6 | 32 ± 4 | 28 ± 5 | 27 ± 4 | 28 ± 4 | 26 ± 7 | 29 ± 8 | 27 ± 7 | 24 ± 6 | 24 ± 4 |  |  |  |
|  | 0.86 | >0.99 | >0.99 | 0.99 | >0.99 | >0.99 | 0.99 | >0.99 | >0.99 | >0.99 | 0.99 | >0.99 | >0.99 | >0.99 |  |  |  |
| **CVP (mmHg)** |  |  |  |  |  |  |  |  |  |  |  |  |  |  |  |  |  |
| Senicapoc | 7 ± 2 | 6 ± 2 | 8 ± 1 | 7 ± 1 | 7 ± 2 | 6 ± 2 | 6 ± 2 | 6 ± 2 | 5 ± 2 | 5 ± 2 | 5 ± 2 | 5 ± 2 | 4 ± 2 | 4 ± 2 | **<0.05** | **<0.05** | **<0.05** |
| Placebo | 7 ± 2 | 7 ± 3 | 9 ± 3 | 8 ± 2 | 8 ± 2 | 8 ± 1 | 8 ± 2 | 8 ± 2 | 8 ± 2 | 7 ± 2 | 7 ± 3 | 8 ± 3 | 8 ± 3 | 7 ± 2 |  |  |  |
|  | >0.99 | 0.97 | 0.99 | 0.99 | 0.99 | 0.47 | 0.67 | 0.18 | 0.08 | 0.49 | 0.50 | 0.16 | 0.09 | 0.53 |  |  |  |

| **Table S2 continued: Hemodynamic and metabolic parameters** | | | | | | | | | | | | | | | **ANOVA (*P* =)** | | |
| --- | --- | --- | --- | --- | --- | --- | --- | --- | --- | --- | --- | --- | --- | --- | --- | --- | --- |
|  | Baseline | Post-injury | 30 min | 60 min | 90 min | 120 min | 150 min | 180 min | 210 min | 240 min | 270 min | 300 min | 330 min | 360 min | Group x Time | Time | Group |
| **Arterial pH** |  |  |  |  |  |  |  |  |  |  |  |  |  |  | 0.86 | **<0.05** | 0.29 |
| Senicapoc | 7,47  ± 0.03 | 7,36 ± 0.11 | 7,10 ± 0,12 | 7,05 ± 0,12 | 7,03 ± 0,13 | 7,07 ± 0.08 | 7.09 ± 0,08 | 7,10 ± 0,10 | 7,12 ± 0,08 | 7,12 ± 0,09 | 7,12 ± 0,09 | 7,13 ± 0,10 | 7,14 ± 0,11 | 7,15 ±  0,12 |  |  |  |
| Placebo | 7,46 ± 0,04 | 7,39 ± 0,08 | 7,11 ± 0,07 | 7,11 ± 0.09 | 7,10 ± 0,09 | 7,11 ± 0,11 | 7,12 ±  0,13 | 7,13 ±  0,15 | 7,17 ±  0,11 | 7,18 ± 0,11 | 7,20 ±  0,11 | 7,21 ± 0,12 | 7,22 ±  0,13 | 7,23 ± 0,12 |  |  |  |
|  | 0.96 | >0.99 | >0.99 | 0.99 | 0.97 | 0.99 | >0.99 | >0.99 | 0.99 | 0.99 | 0.82 | 0.96 | 0.97 | 0.96 |  |  |  |
| **Glucose (mmol/L)** |  |  |  |  |  |  |  |  |  |  |  |  |  |  | 0.44 | **<0.05** | 0.30 |
| Senicapoc | 4,9 ± 0,4 | 6,3 ± 2,7 | 6,7 ± 1,2 | 6,9 ± 1,4 | 6,7 ± 1,9 | 6,1 ± 2,2 | 5,7 ± 1,8 | 5,3 ± 1,5 | 4,7 ± 1,2 | 5,4 ± 1,0 | 4,9 ± 1,2 | 4,6 ± 0,9 | 4,6 ± 0,8 | 4,2 ± 0,7 |  |  |  |
| Placebo | 5,1 ± 0,9 | 5,2 ± 1,0 | 6,2 ± 1,1 | 6.0 ± 1,5 | 5,9 ± 1,6 | 5,6 ± 1,5 | 5,2 ± 1,1 | 5,2 ± 0,9 | 4,9 ± 0,9 | 4,9 ± 0,8 | 4,5 ± 0,9 | 4,6 ± 0,8 | 4,7 ± 0,9 | 4,6 ± 0,7 |  |  |  |
|  | >0.99 | 0.61 | 0.99 | 0.99 | 0.99 | >0.99 | >0.99 | >0.99 | >0.99 | 0.99 | 0.99 | >0.99 | >0.99 | 0.97 |  |  |  |
| **Lactate (mmol/L)** |  |  |  |  |  |  |  |  |  |  |  |  |  |  | 0.90 | **<0.05** | 0.42 |
| Senicapoc | 1,0 ± 0,3 | 2,0 ± 1,3 | 1,5 ± 1,1 | 1,1 ± 0,8 | 0,9 ± 0,5 | 1,0 ± 0,4 | 1,0 ± 0,5 | 1,0 ± 0,5 | 0,8 ± 0,5 | 0,7 ± 0,4 | 0,6 ± 0,3 | 0,6 ± 0,2 | 0,5 ± 0,2 | 0,5 ± 0,3 |  |  |  |
| Placebo | 0,9 ± 0,3 | 1,4 ± 2,0 | 1,0 ± 1,3 | 0,8 ± 0,8 | 0,7 ± 0,5 | 0,7 ± 0,3 | 0,6 ± 0,3 | 0,7 ± 0,4 | 0,7 ± 0,5 | 0,8 ± 0,6 | 0,7 ± 0,7 | 0,7 ± 0,6 | 0,6 ± 0,5 | 0,6 ± 0,4 |  |  |  |
|  | 0.99 | >0.99 | 0.99 | 0.99 | 0.99 | 0.85 | 0.87 | 0.99 | >0.99 | >0.99 | >0.99 | >0.99 | >0.99 | >0.99 |  |  |  |

Data is presented as mean ± standard deviation. Listed P values according to repeated-measurements ANOVA are shown to the right. Results of between group tests are shown below each time point. A *P*<0.05 was considered statistically significant.
