# Supplementary material for: Treatment with senicapoc in a porcine model of acute respiratory distress syndrome
Source: Intensive Care Med Exp. 2021 Apr 19;9:20. doi: 10.1186/s40635-021-00381-z (PMC8053424; doi:10.1186/s40635-021-00381-z)
Supplement: Supplementary file 7 — Additional file 7. [file 40635_2021_381_MOESM7_ESM.docx]

| **Table S3: Respiratory parameters** | | | | | | | | | | | | | | | **ANOVA (*P* =)** | | |
| --- | --- | --- | --- | --- | --- | --- | --- | --- | --- | --- | --- | --- | --- | --- | --- | --- | --- |
|  | Baseline | Post-injury | 30 min | 60 min | 90 min | 120 min | 150 min | 180 min | 210 min | 240 min | 270 min | 300 min | 330 min | 360 min | Group x Time | Time | Group |
| **Respiratory rate** |  |  |  |  |  |  |  |  |  |  |  |  |  |  | 0.39 | **<0.05** | 0.25 |
| Senicapoc | 13 ± 2 | 20 ± 11 | 23 ± 6 | 27 ± 6 | 28 ± 7 | 29 ± 6 | 28 ± 9 | 31 ± 3 | 32 ± 7 | 33 ± 3 | 33 ± 2 | 34 ± 2 | 34 ± 2 | 34 ± 2 |  |  |  |
| Placebo | 15 ± 4 | 17 ± 9 | 24 ± 7 | 24 ± 5 | 27 ± 5 | 28 ± 4 | 28 ± 4 | 28 ± 5 | 29 ± 5 | 29 ± 5 | 29 ± 5 | 31 ± 4 | 31 ± 4 | 29 ± 6 |  |  |  |
|  | 0.82 | >0.99 | >0.99 | 0.99 | >0.99 | >0.99 | >0.99 | 0.93 | 0.97 | 0.74 | 0.69 | 0.72 | 0.72 | 0.59 |  |  |  |
| **Minute volume (mL/min)** |  |  |  |  |  |  |  |  |  |  |  |  |  |  | 0.58 | 0.36 | 0.36 |
| Senicapoc | 5.4 ± 0.8 | 13 ± 3.9 | 5.1 ± 1.2 | 6.1 ± 1.4 | 6.6 ± 1.6 | 6.5 ± 1.9 | 6.6 ± 2.1 | 7.7 ± 1.0 | 8.2 ± 1.0 | 7.7 ± 1.2 | 7.7 ± 1.5 | 8.1 ± 2.1 | 7.5 ± 1.4 | 7.5 ± 1.5 |  |  |  |
| Placebo | 6.4 ± 1,6 | 10 ± 4.7 | 5.8 ± 1.5 | 6.4 ± 1.5 | 7.0 ± 1.8 | 7.1 ± 1.7 | 7.0 ± 1.8 | 7.0 ± 1.7 | 7.0 ± 1.8 | 7.0 ± 1.8 | 7.2 ± 2.3 | 7.2 ± 2.4 | 7.2 ± 2.3 | 6.8 ± 2.2 |  |  |  |
|  | 0.89 | 0.99 | 0.99 | >0.99 | 0.99 | 0.99 | 0.99 | 0.99 | 0.85 | 0.99 | >0.99 | 0.99 | >0.99 | >0.99 |  |  |  |
| **Dynamic compliance (mL/cmH_2_O)** |  |  |  |  |  |  |  |  |  |  |  |  |  |  | 0.06 | **<0.05** | 0.31 |
| Senicapoc | 4.6 ± 1.1 | 4.2 ± 0.8 | 5.3 ± 0.8 | 6.5 ± 1.3 | 6.5 ± 1.1 | 6.1 ± 1.7 | 5.8 ± 1.6 | 5.8 ±  1.6 | 6.1 ±  1.7 | 6.2 ± 1.7 | 5.9 ± 2.0 | 6.4 ± 2.1 | 6.1 ± 2.2 | 6.3 ± 2.4 |  |  |  |
| Placebo | 4.0 ± 1.0 | 3.2 ± 0.6 | 4.4 ± 1.0 | 5.0 ±  0.8 | 5.6 ± 1.0 | 5.7 ± 0.7 | 5.7 ± 0.7 | 5.6 ± 0.9 | 5.3 ± 0.9 | 5.4 ± 1.2 | 5.6 ± 1.1 | 5.7 ± 1.3 | 5.9 ± 1.3 | 5.6 ± 1.1 |  |  |  |
|  | >0.99 | >0.99 | >0.99 | >0.99 | 0.99 | 0.99 | 0.99 | 0.99 | 0.99 | 0.94 | 0.55 | 0.92 | 0.84 | 0.58 |  |  |  |

| **Table S3 continued: Respiratory parameters** | | | | | | | | | | | | | | | **ANOVA (*P* =)** | | |
| --- | --- | --- | --- | --- | --- | --- | --- | --- | --- | --- | --- | --- | --- | --- | --- | --- | --- |
|  | Baseline | Post-injury | 30 min | 60 min | 90 min | 120 min | 150 min | 180 min | 210 min | 240 min | 270 min | 300 min | 330 min | 360 min | Group x Time | Time | Group |
| **Peak inspiratory pressure (cmH_2_O)** |  |  |  |  |  |  |  |  |  |  |  |  |  |  | 0.77 | **<0.05** | 0.19 |
| Senicapoc | 85 ±  14 | 76 ± 13 | 100 ± 23 | 105 ± 29 | 102 ± 33 | 86 ± 19 | 80 ± 19 | 77 ± 17 | 73 ± 20 | 74 ± 14 | 72 ± 16 | 70 ± 19 | 68 ± 18 | 69 ± 18 |  |  |  |
| Placebo | 83 ±  19 | 74 ± 10 | 100 ± 15 | 96 ± 19 | 94 ± 18 | 91 ± 21 | 87 ± 17 | 82 ± 6 | 76 ± 10 | 72 ± 12 | 73 ± 12 | 70 ± 10 | 70 ± 12 | 68 ± 9 |  |  |  |
|  | >0.99 | 0.99 | >0.99 | 0.90 | 0.89 | 0.38 | 0.38 | 0.74 | 0.99 | 0.97 | >0.99 | >0.99 | >0.99 | >0.99 |  |  |  |
| **Mixed venous oxygen saturation (%)** |  |  |  |  |  |  |  |  |  |  |  |  |  |  | 0.34 | **<0.05** | 0.33 |
| Senicapoc | 61 ± 10 | 52 ± 16 | 59 ± 7 | 64 ± 5 | 65 ± 9 | 63 ± 8 | 62 ± 13 | 65 ± 12 | 64 ± 12 | 65 ± 13 | 67 ± 15 | 66 ± 15 | 66 ± 15 | 66 ± 15 |  |  |  |
| Placebo | 55 ± 9 | 46 ± 13 | 56 ± 9 | 61 ± 9 | 60 ± 13 | 58 ± 11 | 59 ± 11 | 58 ± 14 | 58 ± 13 | 58 ± 14 | 58 ± 12 | 57 ± 13 | 63 ± 12 | 63 ± 12 |  |  |  |
|  | 0.93 | 0.99 | 0.99 | 0.99 | 0.99 | 0.99 | >0.99 | 0.99 | 0.99 | 0.99 | 0.94 | 0.97 | >0.99 | >0.99 |  |  |  |
| **Fraction of inspired oxygen** |  |  |  |  |  |  |  |  |  |  |  |  |  |  | **<0.05** | **<0.05** | 0.06 |
| Senicapoc | 0.40 ± 0 | 1 ± 0 | 1 ± 0 | 0.92 ± 0.04 | 0.81 ± 0.09 | 0.78 ± 0.12 | 0.70 ± 0.16 | 0.67 ± 0.18 | 0.64 ± 0.18 | 0.62 ± 0.19 | 0.59 ± 0.21 | 0.58 ± 0.19 | 0.50 ± 0.19 | 0.57 ± 0.19 |  |  |  |
| Placebo | 0.40 ± 0 | 1 ± 0 | 1 ± 0 | 0.98 ± 0.05 | 0.93 ± 0.10 | 0.89 ± 0.09 | 0.85 ± 0.11 | 0.83 ± 0.13 | 0.79 ± 0.14 | 0.75 ± 0.11 | 0.74 ± 0.09 | 0.73 ± 0.09 | 0.70 ± 0.12 | 0.68 ± 0.14 |  |  |  |
|  | - | - | - | 0.37 | 0.39 | 0.59 | 0.43 | 0.56 | 0.73 | 0.83 | 0.74 | 0.68 | 0.94 | 0.96 |  |  |  |

| **Table S3 continued: Respiratory parameters** | | | | | | | | | | | | | | | **ANOVA (*P* =)** | | |
| --- | --- | --- | --- | --- | --- | --- | --- | --- | --- | --- | --- | --- | --- | --- | --- | --- | --- |
|  | Baseline | Post-injury | 30 min | 60 min | 90 min | 120 min | 150 min | 180 min | 210 min | 240 min | 270 min | 300 min | 330 min | 360 min | Group x Time | Time | Group |
| **Partial pressure of carbon dioxide (kPa)** |  |  |  |  |  |  |  |  |  |  |  |  |  |  | 0.12 | **<0.05** | 0.11 |
| Senicapoc | 5.6 ± 0.2 | 6.3 ± 0.4 | 12.5 ± 0.7 | 11.5 ± 0.6 | 12.3 ± 0.7 | 11.6 ± 0.5 | 11.8 ± 0.7 | 11.7 ± 0.8 | 11.7 ± 1.1 | 10.8 ± 1.4 | 11.0 ± 0.9 | 10.5 ± 0.9 | 10.5 ± 1.2 | 10.8 ± 1.1 |  |  |  |
| Placebo | 5.5 ± 0.4 | 6.9 ± 1.7 | 10.8 ± 2.7 | 11.4 ± 1.1 | 11.7 ± 1.1 | 12.8 ± 0.3 | 12.6 ± 0.6 | 12.2 ± 1.6 | 12.8 ± 1.5 | 11.9 ± 1.8 | 13.2 ± 1.8 | 12.8 ± 1.5 | 12.7 ± 1.4 | 12.5 ± 1.8 |  |  |  |
|  | >0.99 | 0.99 | 0.99 | >0.99 | >0.99 | 0.67 | 0.99 | >0.99 | 0.99 | >0.99 | 0.75 | 0.64 | 0.90 | 0.97 |  |  |  |
| **Tidal volume (mL/kg)** |  |  |  |  |  |  |  |  |  |  |  |  |  |  | 0.99 | **<0.05** | 0.44 |
| Senicapoc | 10.1 ± 0.7 | 16.3 ± 6.0 | 5.6 ± 1.3 | 5.5 ± 1.2 | 5.4 ± 1.1 | 5.2 ± 1.1 | 5.4 ± 0.9 | 5.8 ± 1.1 | 5.8 ± 1.1 | 5.5 ± 1.0 | 5.1 ± 1.1 | 5.1 ± 1.2 | 5.0 ± 1.0 | 4.9 ± 0.9 |  |  |  |
| Placebo | 10.0 ± 0.1 | 15.8 ± 6.6 | 6.2 ± 1.9 | 6.4 ± 1.8 | 6.3 ± 1.8 | 6.0 ± 1.5 | 6.1 ± 1.7 | 6.1 ± 1.8 | 6.1 ± 1.8 | 6.0 ± 2.0 | 6.2 ± 2.0 | 5.8 ± 1.9 | 5.8 ± 1.9 | 5.9 ± 1.9 |  |  |  |
|  | >0.99 | >0.99 | 0.99 | 0.97 | 0.98 | 0.98 | 0.99 | >0.99 | >0.99 | >0.99 | 0.97 | 0.99 | 0.99 | 0.97 |  |  |  |

Data is presented as mean ± standard deviation. Listed P values according to repeated-measurements ANOVA are shown to the right. Results of between group tests are shown below each time point. A *P*<0.05 was considered statistically significant.
